# Subtype-specific heterogeneity of myeloid-derived suppressor cells in breast cancer: current insights and future directions

**DOI:** 10.7717/peerj.20937

**Published:** 2026-04-13

**Authors:** Biyao Gong, Lixiang Zheng

**Affiliations:** Jiangxi University of Traditional Chinese Medicine, NanChang, China

**Keywords:** Breast cancer, MDSCs, TME, Molecular subtypes, Immunosuppression

## Abstract

Breast cancer remains the most prevalent malignancy among women worldwide, and its molecular subtypes display marked differences in clinical outcomes and therapeutic responses. Increasing evidence highlights the critical roles of myeloid-derived suppressor cells (MDSCs) within the tumor microenvironment, where they orchestrate immune evasion, promote metastasis, and contribute to therapy resistance. However, most current studies primarily focus on triple-negative breast cancer (TNBC), while systematic insights into the abundance, subset distribution, and functional heterogeneity of MDSCs in Luminal and HER2^+^ subtypes remain limited. This review synthesizes recent advances on major MDSC programs and highlights subtype-associated differences in their distribution patterns, immunosuppressive mechanisms, and clinical relevance across TNBC, HER2^+^, and HR^+^/Luminal breast cancer. We further discuss how these subtype-linked myeloid states may influence key therapeutic outcomes, including pathological complete response following neoadjuvant therapy, heterogeneity of benefit from immunotherapy-based combinations, and the emergence of treatment resistance. In addition, we summarize emerging single-cell and spatial omics approaches that refine MDSC classification and enable *in situ* mapping of myeloid–lymphocyte organization. Finally, we outline mechanism-guided therapeutic strategies targeting MDSCs—encompassing recruitment/trafficking blockade, inhibition of suppressive metabolic effector pathways, and myeloid ecosystem remodeling/reprogramming—to support subtype-tailored precision immunotherapy in breast cancer. This review is intended for researchers in tumor immunology and cancer biology, clinicians and translational medicine professionals, as well as graduate students or early-career scholars interested in immunosuppressive cells and breast cancer.

## Introduction

Breast cancer is the most common malignancy among women worldwide and is characterized by marked heterogeneity (Bray et al., 2024; Perou et al., 2000). Based on molecular profiling, breast cancer can be classified into Luminal A, Luminal B, HER2^+^, and triple-negative breast cancer (TNBC) subtypes (Goldhirsch et al., 2011). In recent years, the tumor microenvironment (TME) has received increasing attention for its critical role in breast cancer initiation and progression (Akinsipe et al., 2024). Among its cellular components, myeloid-derived suppressor cells (MDSCs) represent a key immunosuppressive population that promotes immune evasion, metastasis, and therapeutic resistance through multiple mechanisms, including the secretion of metabolic mediators, cytokines, and the expression of immune checkpoint molecules (Liu et al., 2023; Wang et al., 2025; Weber et al., 2018). Notably, the abundance, subset composition, and functional activity of MDSCs vary across molecular subtypes: TNBC is often associated with heightened immunosuppression, while Luminal and HER2^+^ subtypes can induce MDSC expansion under specific signaling cues or therapeutic pressure (Cha & Koo, 2020; Kumar, Da Silva & Chaves, 2024). Elucidating the characteristics and roles of MDSCs across breast cancer subtypes is therefore of great importance for the development of precision immunotherapeutic strategies.

## Survey Methodology

To ensure comprehensive and unbiased coverage of the literature, we performed a structured search strategy across multiple scholarly databases. The primary databases included PubMed, Web of Science, Scopus, and Google Scholar. Searches were conducted using English keywords and their combinations: “myeloid-derived suppressor cells”, “MDSCs”, “breast cancer subtypes”, “triple-negative breast cancer”, “HER2-positive breast cancer”, “Luminal A”, “Luminal B”, “tumor microenvironment”, “immune suppression”, “phenotypic heterogeneity”, “epigenetic regulation”, “therapy resistance”, and “immunotherapy”. Boolean operators (“AND”, “OR”) were applied to refine search results.

Importantly, specific efforts were made to systematically identify studies addressing Luminal (A/B) and HER2-positive breast cancer subtypes, despite the fact that the majority of MDSC-related studies in breast cancer have historically focused on triple-negative breast cancer (TNBC). All eligible studies explicitly examining MDSCs in Luminal or HER2-positive contexts were included whenever available. The relatively limited number of such studies reflects the current state of the literature rather than a selection bias introduced by the authors.

A preliminary screening was performed to evaluate whether each article addressed the scope of this review, focusing on the abundance, phenotypic profiles, functional mechanisms, and clinical significance of MDSCs in breast cancer. Full-text evaluation was subsequently conducted to assess methodological rigor and the credibility of the reported findings. Both basic experimental studies (*in vitro* and *in vivo*) and clinical/translational research were considered.

Earlier literature reviews and meta-analyses were consulted to ensure that key research themes were not overlooked and to minimize the potential for bias. In addition, reference lists of included studies were manually examined to identify additional relevant publications. This multi-step process ensured comprehensive coverage of current knowledge while maintaining a critical and balanced perspective.

### Molecular subtypes of breast cancer and their clinical significance

Breast cancer remains the most common malignancy among women worldwide, with increasing incidence and mortality rates, particularly in low- and middle-income countries (Arnold et al., 2022; Sung et al., 2021). The four molecular subtypes of breast cancer each exhibit distinct characteristics in hormone receptor expression, proliferative activity, prognosis, and therapeutic response (Coates et al., 2015; Goldhirsch et al., 2011), as shown in Table 1. Notably, different subtypes require distinct prognostic evaluations and treatment strategies. For instance, triple-negative breast cancer (TNBC) is often associated with poor prognosis and limited sensitivity to immunotherapy (Qattan, 2020).

### Definition, classification, and role of myeloid-derived suppressor cells (MDSCs) in tumor immune evasion

#### Immunosuppressive mechanisms and functions of MDSCs in the breast cancer microenvironment

**Figure 1:**
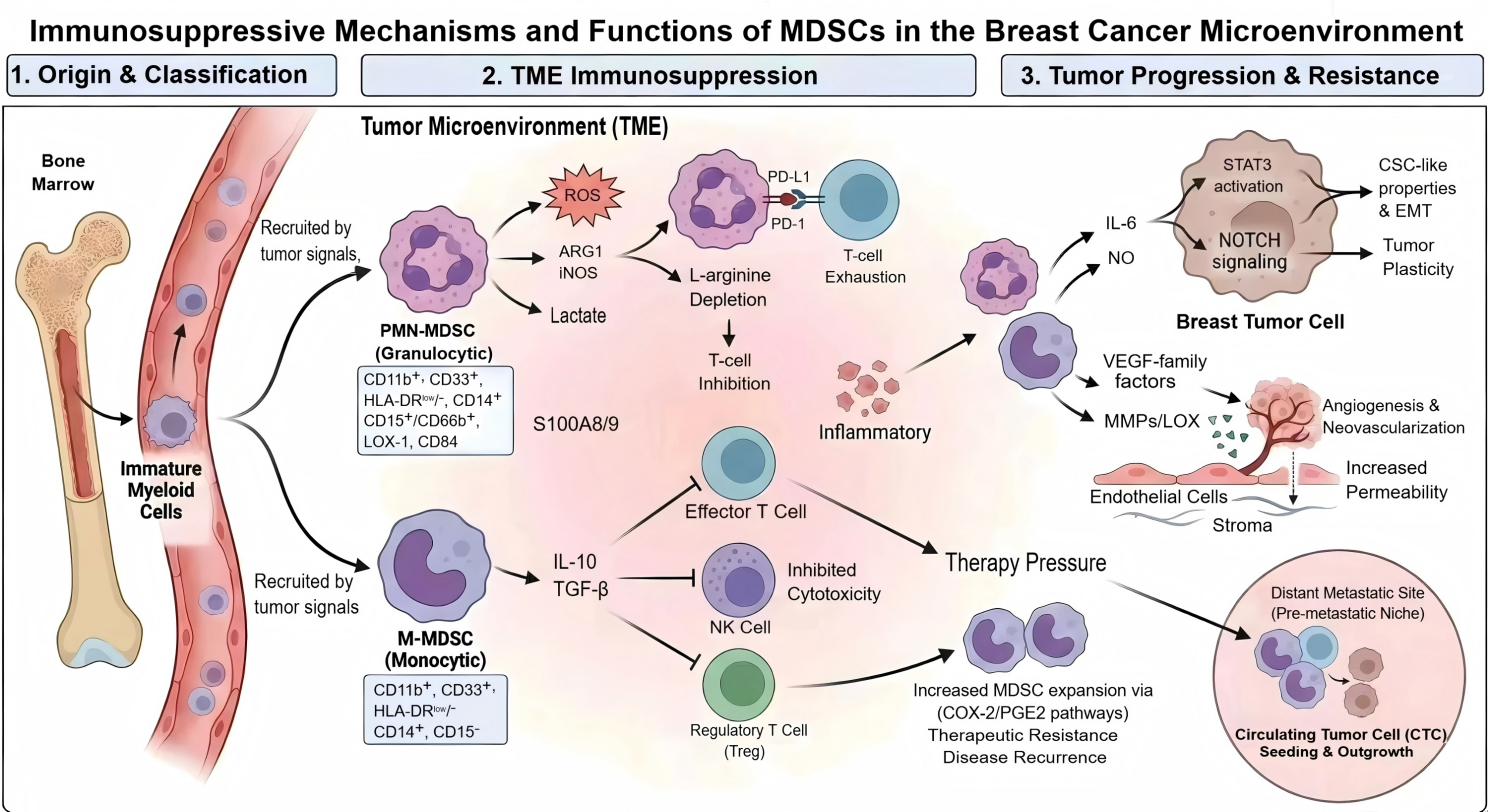
Immunosuppressive mechanisms and functions of MDSCs in the breast cancer microenvironment. Figure created with BioGDP.com.

Despite advances in surgery, radiotherapy, chemotherapy, endocrine therapy, and targeted therapy, long-term outcomes of breast cancer remain hindered by disease recurrence and therapeutic resistance (Salemme et al., 2023). Within the TME, MDSCs play a pivotal role in suppressing anti-tumor immune responses through cytokine secretion and direct cell–cell interactions, thereby promoting tumor growth and contributing to therapeutic resistance (Kundu et al., 2024; Haist et al., 2021). Among these, MDSCs are particularly enriched in breast tumors, where they strongly inhibit T and NK cell activity and secrete pro-tumorigenic factors (Rosado-Sanz et al., 2025), ultimately shaping a microenvironment favorable for tumor growth and dissemination (Fig. 1; Jiang et al., 2025).

**Table 1:** Four molecular subtypes of breast cancer: key features and treatment priorities.

**Subtype**	**Common IHC surrogates**	**Key biological/clinical features**	**Clinical behavior/prognosis**	**Treatment priorities**
Luminal A	ER^+^/PR^+^, HER2^−^, relatively low Ki-67 (Abubakar et al., 2019).	Hormone-driven, lower proliferation, often better differentiated (Yersal & Barutca, 2014)	Generally favorable outcomes	Endocrine therapy as backbone; chemotherapy reserved for higher-risk disease based on integrated risk assessment (Cao et al., 2024)
Luminal B	ER^+^ predominant; HER2^−^ or HER2^+^; higher Ki-67 and/or low PR (Inic et al., 2014).	Higher proliferation and heterogeneity; endocrine-sensitive but more resistant-prone than Luminal A (Denkert et al., 2025; Yersal & Barutca, 2014)	Higher recurrence risk than Luminal A (Inic et al., 2014)	Endocrine therapy plus risk-adapted chemotherapy; if HER2^+^, anti-HER2 therapy is typically incorporated (see footnote) (Pascual et al., 2021)
HER2^+^	HER2 overexpression/amplification (IHC 3^+^ or ISH^+^); HR status variable (often discussed as HER2-positive non-luminal/HER2-enriched in simplified schemes) (Wolff et al., 2018)	HER2-driven, highly proliferative; strong dependence on targeted blockade (Swain, Shastry & Hamilton, 2023)	Aggressive if untreated; markedly improved with modern anti-HER2 therapy (Kreutzfeldt et al., 2020)	Anti-HER2–based systemic therapy (*e.g.*, trastuzumab ±pertuzumab) with chemo and/or other systemic components depending on stage/risk (Von Minckwitz et al., 2017)
TNBC	ER^−^/PR^−^/HER2^−^	Often basal-like features; early relapse/metastasis risk; heterogeneous with immunotherapy-responsive subsets (Juan & Peng, 2025)	Overall poorer prognosis with substantial heterogeneity (Dogra et al., 2025)	Chemotherapy backbone; immunotherapy (PD-1/PD-L1) in selected settings; PARP inhibitors for eligible molecular contexts (Lebedeva et al., 2025)

MDSCs represent a heterogeneous population of immature myeloid cells originating from the bone marrow, which accumulate extensively in diverse malignancies and exert potent immunosuppressive activity through multiple mechanisms (Li et al., 2021a). Specifically, MDSCs secrete metabolic mediators such as arginase-1 (Arg1), inducible nitric oxide synthase (iNOS), and reactive oxygen species (ROS) to deplete metabolites essential for T-cell function (Miret et al., 2019). They also release immunoregulatory cytokines including IL-10 and TGF-*β* to expand regulatory T cells, while expressing inhibitory checkpoint molecules such as PD-L1 to attenuate immune responses (Grzywa et al., 2020; Ibrahim et al., 2025). In breast cancer, both monocytic MDSCs (M-MDSCs) and polymorphonuclear MDSCs (PMN-MDSCs) are recruited by tumor-derived signals and accumulate in primary tumors as well as metastatic sites (Karin, 2020). This leads to the establishment of a “pre-metastatic niche” enriched in MDSCs, which remodels the local microenvironment and creates a permissive condition for circulating tumor cell seeding and outgrowth (Gabrilovich, Ostrand-Rosenberg & Bronte, 2012).

Importantly, these canonical suppressive programs are supported by breast cancer–specific clinical and mechanistic evidence. Clinically, circulating MDSC frequencies increase with advanced stage and metastatic burden, change dynamically with treatment exposure (including neoadjuvant chemotherapy), and associate with unfavorable outcomes in advanced breast cancer cohorts (Gonda et al., 2017; Saed et al., 2024). Mechanistically, studies in breast cancer models indicate that MDSCs contribute directly to tumor aggressiveness by promoting tumor cell plasticity and immune escape. In particular, MDSCs have been shown to enhance cancer stem cell (CSC)-like properties *via* IL-6/STAT3 activation and NO-dependent NOTCH signaling, and myeloid suppressor populations can facilitate EMT-related programs and metastatic progression in murine breast cancer systems (Peng et al., 2016; Zhu et al., 2017). Beyond immune suppression, MDSCs also facilitate vascular and stromal remodeling: they release pro-angiogenic mediators (*e.g.*, VEGF-family factors) and matrix-remodeling enzymes (*e.g.*, MMPs/LOX), thereby increasing endothelial activation and permeability, supporting neovascularization, and lowering barriers to tumor cell extravasation and outgrowth at distant sites (Hsu et al., 2019; Liu & Zhang, 2025; Zhang et al., 2020). These effects are further reinforced by inflammatory mediators such as S100A8/9 and sustained impairment of cytotoxic immune surveillance (T cells/NK cells) (Shou et al., 2016). Moreover, therapy pressure may further amplify these circuits; for example, resistant tumor states have been linked to increased MDSC expansion and suppressive polarization through COX-2/PGE2-associated pathways, providing a rationale for combining standard therapies with myeloid-modulating strategies to mitigate therapy-driven immune resistance (Cuenca-Escalona et al., 2024; Zheng et al., 2025).

#### Phenotypic and molecular characteristics of MDSCs

In addition to differences in abundance across breast cancer subtypes, MDSCs exhibit considerable heterogeneity in their phenotypic and molecular marker expression. Conventionally, MDSCs are classified into two major subpopulations: granulocytic (G-MDSCs, also referred to as PMN-MDSCs) and monocytic (M-MDSCs) (Bergenfelz & Leandersson, 2020). In humans, M-MDSCs are typically defined as CD11b^+^CD33^+^HLA-DR^low^/–CD14^+^CD15^−^, whereas G-MDSCs are characterized as CD11b^+^CD33^+^HLA-DR^low^/–CD14^−^CD15^+^ (or CD66b^+^) (Bronte et al., 2016; Liu et al., 2023), as shown in Table 2. However, these conventional markers largely overlap with other myeloid lineages, limiting their specificity. Recent advances in single-cell transcriptomics and high-dimensional cytometric profiling have provided a more systematic understanding of the heterogeneity of tumor-associated myeloid cells and identified more specific molecular markers for MDSCs. Representative examples include CD84 (identified as an MDSC-specific surface marker in murine breast cancer models through single-cell RNA sequencing, which improves detection efficiency when combined with CD11b/Gr-1 or CD33/CD11b/HLA-DR (Alshetaiwi et al., 2020)), and LOX-1/OLR1 (validated through transcriptomic, flow cytometric, and functional analyses (Condamine et al., 2016)). Subsequent studies have proposed additional candidates, such as CD52, PTGER2, and combinations of CD10/CD16 as markers for identifying mature PMN-MDSCs (Pettinella et al., 2024).

**Table 2:** Phenotypic characteristics and functional features of major MDSC subsets in breast cancer.

**MDSC subtype**	**Abundance**	**Marker for basic subtype classification**	**Marker-specific description**	**Function**	**Phenotypic features**
PMN-MDSC	High in peripheral blood	CD11b+ CD14- CD15+ (or CD66b+), HLA-DR- (Bronte et al., 2016)	CD52, PTGER2, CD10/CD16:Markers used for the identification of mature PMN-MDSCs LOX-1 (OLR1) can be used to distinguish between PMN-MDSCs and mature neutrophils (Condamine et al., 2016; Pettinella et al., 2024).	T-cell suppression *via* ROS generation, metabolic depletion, and immune checkpoint regulation (*e.g.*, PD-L1) (Li et al., 2021c; Wang et al., 2025)	Granulocytic, high ROS production, oxidative metabolism activity (Wang et al., 2025)
M-MDSC	Moderate in tumor tissue	CD11b+ CD14+ HLA-DR- CD15- (Bronte et al., 2016)	No subtype-specific markers available in current research	T-cell suppression *via* cytokine secretion (IL-10, TGF-*β*), immune checkpoint expression (PD-L1), and tissue remodeling (Gneo et al., 2022; Groth et al., 2019)	Monocytic, high secretion of immunosuppressive cytokines, involved in immune microenvironment modulation (He, Zheng & Qi, 2025)

Collectively, these findings emphasize that no single marker can serve as an “absolute” diagnostic indicator, as expression of molecules such as LOX-1 can also be induced by inflammation, G-CSF treatment, or infection. Moreover, the expression of these markers displays plasticity and heterogeneity across different breast cancer subtypes. Therefore, the optimal approach is to employ single-cell–defined markers (*e.g.*, LOX-1 or CD84) for enrichment or preliminary identification, followed by validation using conventional granulocytic/monocytic markers (CD15/CD66b or CD14/HLA-DR) and functional assays (*e.g.*, T-cell suppression).

#### Differential roles of PMN-MDSCs and M-MDSCs

Despite sharing an immunosuppressive role, PMN-MDSCs and M-MDSCs display distinct functional specializations, mechanistic pathways, and spatiotemporal distributions within the breast cancer microenvironment (Marvel & Gabrilovich, 2015). PMN-MDSCs primarily suppress T cell function by releasing reactive oxygen species (ROS) and arginase-1 (ARG1), which deplete L-arginine, a key metabolite required for T cell activity (Wang et al., 2025). The elevated expression of ARG1 not only inhibits T cell proliferation and effector functions but also creates an immunosuppressive environment that facilitates tumor immune escape (Yang et al., 2020). Studies suggest that PMN-MDSCs may also contribute to the formation of an immune-suppressive environment through the secretion of metabolic products, such as lactate (Choi et al., 2025). Additionally, PMN-MDSCs further suppress T cell immunity by upregulating immune checkpoint molecules, such as PD-L1, thereby limiting the activity of tumor-specific T cells (Li et al., 2021c).

In contrast to PMN-MDSCs, M-MDSCs primarily exert their immunosuppressive effects by secreting cytokines such as IL-10 and TGF-*β*, which inhibit the activity of effector T cells (Gneo et al., 2022; Groth et al., 2019). These cytokines not only suppress T cell proliferation, cytokine production, and cytotoxic function but also modulate the immune response within the tumor microenvironment, promoting immune escape and resistance to therapy (He, Zheng & Qi, 2025).

While both PMN-MDSCs and M-MDSCs contribute to immune suppression, they do so through distinct mechanisms. PMN-MDSCs predominantly rely on metabolic pathways and ROS production to inhibit T cell function, whereas M-MDSCs are more dependent on the secretion of cytokines and cell-to-cell interactions to regulate the immune microenvironment. These differential mechanisms enable them to play distinct roles in tumor immune evasion and the development of therapeutic resistance (Grzywa et al., 2020; He, Zheng & Qi, 2025).

### Subtype-specific differences in MDSCs across breast cancer molecular classifications

#### Differences in the abundance of MDSCs across breast cancer molecular subtypes

In recent years, studies investigating the distribution of MDSCs have primarily focused on three aspects: overall abundance, the relative composition of polymorphonuclear MDSCs (PMN-MDSCs) *versus* monocytic MDSCs (M-MDSCs), and the heterogeneity of their localization in peripheral blood *versus* tumor-infiltrating compartments. These key findings are summarized in Table 3.

**Table 3:** Comparison of MDSC abundance and immunosuppressive functions across the four main molecular subtypes of breast cancer.

**Breast cancer subtype**	**MDSC abundance**	**Immunosuppressive functions**
Luminal A	Low	Moderate T-cell suppression, immune checkpoint regulation, limited MDSC expansion (Liu et al., 2023)
Luminal B	Low	Increased T-cell suppression, immune checkpoint expression (PD-L1), therapy-induced MDSC expansion (Moura et al., 2025)
HER2-positive	Intermediate	Increased immune suppression, CXCR2 axis activation, therapeutic resistance (Korbecki et al., 2025)
Triple-negative (TNBC)	High	Strong immune evasion, pre-metastatic niche formation, enhanced cancer stem cell traits (Yang & Wang, 2025)

Several investigations have compared immune cell infiltration within the TME of different breast cancer molecular subtypes. Overall, TNBC and HER2^+^ subtypes display higher levels of immune infiltration compared with ER^+^ subtypes (Loi, 2013; Otterlei Fjørtoft, Huse & Rye, 2024). Specifically, a study published in The Journal of Clinical Investigation reported that TNBC patients exhibit significantly elevated MDSC levels compared with non-TNBC patients (Kumar et al., 2018). Similarly, a separate review highlighted that human TNBC tumors are characterized by increased MDSC abundance, with a strong association between MDSC levels and TNBC progression (Liu et al., 2023). By contrast, ER^+^ subtypes are generally marked by lower immune cell infiltration and correspondingly reduced MDSC levels (Otterlei Fjørtoft, Huse & Rye, 2024). Evidence regarding the HER2^+^ subtype is relatively limited. However, HER2^+^ tumor cells have been shown to secrete chemokines such as TGF-*β* and CCL22, which can recruit both Tregs and MDSCs into the TME (Perrone et al., 2021). Notably, ARG1^+^ myeloid cells—including MDSCs—were found to be highly enriched across ER^+^, HER2^+^, and TNBC tumors, suggesting that the breast cancer microenvironment as a whole tends to promote MDSC activation (Su et al., 2021).

In summary, current evidence indicates that MDSC abundance is highest in TNBC, intermediate in HER2^+^ tumors, and lowest in ER^+^ subtypes, consistent with the overall immune profiles of these categories. However, methodological differences across studies—including flow cytometry, immunohistochemistry, and peripheral blood analysis—introduce variability, and most investigations to date have concentrated on TNBC or breast cancer as a whole rather than systematically comparing all subtypes. Future studies are therefore warranted to conduct stratified and stage-specific analyses of MDSC abundance, subset composition, and functional states across different breast cancer molecular subtypes.

With respect to the heterogeneity of distribution between peripheral blood and tumor-infiltrating sites, peripheral blood is predominantly enriched in PMN-MDSCs, whereas tumors tend to harbor more M-MDSCs, which also exhibit stronger suppressive activity (Bergenfelz et al., 2015; Cha & Koo, 2020; He, Zheng & Qi, 2025). This suggests that the composition and function of MDSCs may differ across anatomical sites within the same patient. Among different breast cancer subtypes, as noted above, TNBC patients are particularly prone to MDSC enrichment both in circulation and within the tumor. Mechanistic studies indicate that TNBC/basal-like tumors upregulate factors such as ΔNp63 and CCL20, thereby promoting myeloid progenitor differentiation toward the granulocytic lineage and recruiting PMN-MDSCs, leading to their expansion in both the peripheral circulation and the tumor microenvironment (Kumar et al., 2018; Zhang et al., 2023). Comparative analyses of MDSCs between TNBC and other breast cancer subtypes generally support the trend that peripheral PMN-MDSC levels are higher in TNBC, as demonstrated by several prospective studies (Tan et al., 2022); however, more rigorous and systematic investigations are warranted to establish this association conclusively. At the tumor-infiltration level, TNBC/basal-like tumors display a higher abundance of MDSCs than non-TNBC, facilitating immune evasion and disease progression (Kim et al., 2024). Taken together, the preferential enrichment of PMN-MDSCs in peripheral blood and M-MDSCs within tumors—representing “site-specificity”—together with the “subtype-specificity” of TNBC, collectively shape a profoundly immunosuppressive landscape and contribute to the poorer clinical outcomes observed in TNBC patients.

#### Alterations of MDSCs across the four molecular subtypes

In TNBC, a pronounced enrichment of PMN-MDSCs is observed more frequently than in other subtypes. These cells not only increase markedly in number but are also accompanied by robust activation of the CXCR2 axis—including ligands such as CXCL8/IL-8, CXCL1, CXCL2, and CXCL5—which facilitates their efficient recruitment into the tumor microenvironment (Bullock & Richmond, 2021; Dominguez et al., 2017). Concurrently, the metabolic stress and hypoxic conditions that are pervasive in the TNBC microenvironment further drive the immunosuppressive polarization of MDSCs, characterized by elevated expression of arginase-1 (ARG1), reactive oxygen species (ROS), and the immune checkpoint molecule PD-L1 (Lu et al., 2024). Collectively, these alterations establish a uniquely immunosuppressive milieu in TNBC, conferring potent T-cell suppression and enhanced immune evasion capacity (Imani et al., 2025). By contrast, HER2^+^ and HR^+^/Luminal subtypes generally exhibit lower overall levels of myeloid suppressor cell infiltration compared to TNBC (Hammerl et al., 2018). Nevertheless, MDSC accumulation is not absent in these subtypes but instead appears to depend more heavily on specific signaling cues and environmental contexts. For instance, sustained activation of the IL-6/STAT3 pathway, hypoxia-driven HIF-1*α* signaling, and therapy-induced stress from radiotherapy, chemotherapy, or anti-HER2 targeted treatment can induce the expansion and functional reprogramming of M-MDSCs and PMN-MDSCs (Peng et al., 2016; Vasquez-Dunddel et al., 2013). Under such conditions, MDSCs frequently manifest as HLA-DR^low^/–CD14^+^ M-MDSCs or CD15^+^/LOX-1^+^ PMN-MDSCs, often with concomitant upregulation of PD-L1, ARG1, or iNOS, thereby compensating for their otherwise lower baseline immunosuppressive capacity (Wu et al., 2017).

In HER2^+^ breast cancer, the biological features of MDSCs have not yet been systematically characterized; however, available evidence suggests a potential “HER2–myeloid crosstalk” (Choi et al., 2025). HER2 overexpression can induce tumor cells to secrete pro-inflammatory cytokines such as IL-6 and IL-8 and to activate NF-*κ*B and IL-6/STAT3 signaling, while simultaneously releasing GM-CSF, G-CSF, TGF-*β* and CCL22 (Huang et al., 2025; Raftery et al., 2025). These factors may promote the differentiation of immature myeloid progenitors into MDSCs and drive their co-recruitment with regulatory T cells into the tumor microenvironment. On this basis, it is plausible that, under conditions of sustained HER2 signaling and therapy-induced stress, MDSCs amplify inflammatory and stemness-related pathways and thereby attenuate responses to anti-HER2 monoclonal antibodies or combinatorial immunotherapies. This putative “HER2–MDSC amplification loop” remains hypothetical and requires further validation in HER2^+^-enriched clinical cohorts and experimental models.

HR^+^/Luminal disease may thus represent a state with low baseline myeloid infiltration but heightened sensitivity to inflammation- and therapy-driven MDSC expansion. The precise links between MDSCs, endocrine resistance and late recurrence in Luminal breast cancer remain to be elucidated in molecular subtype–stratified clinical and translational studies (Liu et al., 2023). Overall, the most significant gap across both subtypes is the lack of subtype-stratified clinical trials. Most current MDSC-targeting strategies are tested in “all-comer” populations or focused solely on TNBC. Future research must prioritize dissecting the distinct metabolic and signaling dependencies of MDSCs in the specific microenvironments of Luminal and HER2+ tumors to develop precision immunotherapies.

Taken together, both quantitative and qualitative differences define MDSC phenotypes and functions across breast cancer subtypes: TNBC is distinguished by a high abundance of strongly immunosuppressive PMN-MDSCs, whereas HER2^+^ and HR^+^/Luminal tumors display comparatively lower baseline levels but can still trigger substantial MDSC expansion and activation under specific microenvironmental signals or therapeutic stress. These subtype-specific differences not only underscore the distinct roles of MDSCs in breast cancer immune evasion but also highlight the need for molecular subtype–tailored strategies when designing MDSC-targeted interventions.

### The role of MDSCs in therapeutic response and resistance in breast cancer

In the context of endocrine therapy (predominantly in Luminal subtypes), prolonged anti-hormonal pressure can induce MDSC accumulation and activate the IL-6/STAT3 axis, thereby impairing CD8^+^ T-cell effector functions and leading to microenvironmental acquired resistance (Tsoi et al., 2021). This suggests that, in refractory or relapsed cases, concomitant inhibition of inflammatory–myeloid pathways should be considered to preserve antitumor immunity. During HER2-targeted therapy (*e.g.*, trastuzumab), MDSCs counteract antibody-dependent cellular cytotoxicity (ADCC) by upregulating PD-L1 and inducing nutrient deprivation through ARG1 activity (Chaganty et al., 2018; Petricevic et al., 2013). This mechanism helps explain why some patients fail to achieve durable benefit despite the mobilization of immune effector cells. Chemotherapy and radiotherapy often reduce MDSCs initially, yet treatment-induced stress can subsequently mobilize bone marrow–derived progenitors, resulting in secondary MDSC enrichment. This, in turn, establishes a pro-inflammatory and immunosuppressive ecosystem that frequently precedes relapse and resistance (Kang et al., 2020). For immune checkpoint blockade (ICB), MDSCs—particularly PMN-MDSCs—serve as a critical barrier to response in TNBC and related subtypes. By restricting CD8^+^ T-cell infiltration and effector function, they markedly reduce the probability of clinical benefit from ICB (Li et al., 2021b; Li et al., 2021c).

These therapy-associated effects display both subtype and site specificity. TNBC is more prone to significant PMN-MDSC accumulation, along with hyperactivation of the CXCR2 axis (CXCL8/IL-8, CXCL1/2/5) and hypoxia-driven immunosuppressive polarization, characterized by elevated ARG1, ROS, and PD-L1 expression (Dominguez et al., 2017; Lazennec, Rajarathnam & Richmond, 2024). This combination creates a microenvironment particularly hostile to T-cell function. In contrast, HER2^+^ and HR^+^/Luminal tumors generally exhibit lower baseline MDSC infiltration, yet remain capable of substantial MDSC expansion and functional reprogramming under IL-6/STAT3 and HIF-1*α* signaling or therapeutic stress (Wang et al., 2018). Moreover, the composition of MDSCs differs between peripheral circulation and the tumor microenvironment: PMN-MDSCs dominate in blood, whereas M-MDSCs are more enriched within tumors and display stronger suppressive activity (Kumar et al., 2016). In TNBC/ER-negative patients, elevated circulating PMN-MDSC levels have been associated with lower pathological complete response (pCR) rates following neoadjuvant therapy (Wesolowski et al., 2017). These findings highlight the potential predictive and translational value of monitoring and targeting MDSCs during perioperative or treatment windows.

### Therapeutic strategies targeting MDSCs to improve precision immunotherapy in breast cancer

Mechanistically, MDSC-directed strategies can be broadly grouped into: blocking recruitment and trafficking into tumors and metastatic niches, attenuating suppressive effector programs—particularly metabolic pathways that constrain T-cell function, and remodeling or reprogramming immunosuppressive myeloid ecosystems (Wang et al., 2025). Among these, breast cancer–relevant evidence is most mature for chemokine-axis recruitment blockade, ARG1-centered metabolic inhibition, and myeloid remodeling/reprogramming approaches, most often implemented as combination modules with immune checkpoint blockade (ICB), chemotherapy, and/or targeted therapy (Liu et al., 2023; O’Connell et al., 2024; Steggerda et al., 2017).

#### Recruitment/trafficking blockade

Chemokine-driven recruitment is especially relevant for PMN-MDSCs. In inflammatory tumor microenvironments—most prominently in a substantial subset of TNBC—CXCR2 ligands (*e.g.*, CXCL1/2/5/8-related networks) can drive PMN-MDSC trafficking and intratumoral accumulation, reinforcing immune suppression (Bullock & Richmond, 2021). CXCR2 antagonism thus provides a direct strategy to reduce PMN-MDSC influx and relieve suppressive pressure on effector lymphocytes, creating a mechanistic rationale for combination with ICB (Zhang et al., 2023). For clinical translation, studies should prioritize pharmacodynamic endpoints that demonstrate decreased tumor PMN-MDSC density, restoration of CD8^+^ T-cell/NK-cell activation signatures, and spatial readouts indicating reduced myeloid exclusion of effector cells (Guo et al., 2023; Pozniak et al., 2025).

#### Functional suppression blockade

Pharmacologic arginase inhibition has shown the ability to restore anti-tumor immune activity in preclinical systems and to enhance responses when combined with PD-1/PD-L1–based regimens, consistent with an “immune unlocking” mechanism (Sosnowska et al., 2021). In breast cancer, ARG1-centered strategies are most appropriately positioned as combination modules, with greatest plausibility in TNBC and in selected HER2^+^ contexts where inflammatory cues and therapy pressure may amplify ARG1-linked suppressive states (Retecki et al., 2021; Su et al., 2021). Key translational priorities include confirming on-target effects on arginine metabolism, clarifying the dominant cellular sources of ARG1 *in situ*, and applying biomarker-driven enrichment based on baseline myeloid abundance and lymphocyte dysfunction.

#### Myeloid ecosystem remodeling and reprogramming

The CSF1–CSF1R axis regulates multiple tumor-associated myeloid populations, and CSF1R-directed strategies can remodel suppressive myeloid ecosystems that overlap phenotypically and functionally with monocytic MDSCs (Sato et al., 2025; Zhu et al., 2014). While conceptually attractive, breast cancer translation highlights that safety and context-dependence can be rate limiting; therefore, CSF1R-based combinations should be advanced only with clear *in situ* evidence of myeloid remodeling and well-defined patient selection criteria (O’Brien et al., 2021). In parallel, reprogramming approaches (*e.g.*, epigenetic modulation such as HDAC inhibition) provide a “state-switch” paradigm that treats MDSCs as plastic and therapeutically programmable, with preclinical evidence supporting synergy with ICB through attenuation of myeloid-mediated suppression and improved immune-permissive states (Christmas et al., 2018). Future work should define minimal effective exposure, identify susceptible myeloid states, and validate whether reprogramming translates into measurable restoration of human effector lymphocyte function.

#### Subtype-tailored implementation and actionable endpoints

Across subtypes, rigorously designed, subtype-stratified trials incorporating harmonized phenotyping (classical markers plus transcriptomic/metabolic signatures), functional suppression assays, and spatially resolved readouts will be essential to establish causality between myeloid remodeling and therapeutic benefit and to enable truly mechanism-informed precision immunotherapy in breast cancer.

## Conclusion

Although evidence on MDSC heterogeneity in breast cancer has expanded rapidly, the field remains uneven and methodologically fragmented across molecular subtypes. Overall, available data support subtype-associated differences in major MDSC programs that extend beyond immune suppression to shape microenvironmental remodeling and treatment responsiveness. TNBC most consistently displays a myeloid-inflamed state with prominent PMN-MDSC–linked suppressive activity; HER2^+^ disease appears more context-dependent, with myeloid suppression often emerging or intensifying under inflammatory cues and therapeutic pressure; and HR^+^/Luminal tumors generally show lower baseline myeloid infiltration but may still develop clinically meaningful MDSC expansion during progression or therapy. Collectively, these subtype-linked myeloid states are poised to influence key therapeutic outcomes, including pCR following neoadjuvant therapy, heterogeneity of benefit from immunotherapy combinations, and the development of resistance, underscoring the need for subtype-stratified myeloid frameworks to enable precision immunotherapy.

To close these gaps, several actionable priorities should be pursued. First, systematic single-cell profiling of Luminal B tumors—ideally coupled to endocrine treatment contexts—will be critical to resolve subtype-specific myeloid programs associated with endocrine resistance and disease progression. Second, spatially resolved mapping in HER2^+^ models and enriched clinical cohorts should define the *in situ* organization of MDSCs relative to CD8^+^ T cells/NK cells under therapy-induced stress, thereby clarifying whether myeloid exclusion or suppression dominates and identifying tractable interaction nodes. Third, harmonized multi-modal phenotyping that integrates flow cytometry with single-cell/spatial transcriptomics, paired blood–tumor sampling, and functional suppression assays is needed to improve cross-study comparability and to link tumor-resident MDSC states with clinically meaningful endpoints. Together, these efforts will sharpen mechanistic inference and accelerate rational incorporation of MDSC-modulating strategies into subtype-tailored therapeutic paradigms.

## Additional Information and Declarations

### Data Availability

The following information was supplied regarding data availability: This is a literature review.
